# Mucinous borderline ovarian tumors: pathological and prognostic study at Salah Azaiez Institute

**DOI:** 10.11604/pamj.2022.41.349.32332

**Published:** 2022-04-29

**Authors:** Ghada Sahraoui, Asma Fitouri, Lamia Charfi, Maha Driss, Maher Slimane, Monia Hechiche, Karima Mrad, Raoudha Doghri

**Affiliations:** 1 Pathology Department, Salah Azaiez Institute, Tunis, Tunisia,; 2 Faculty of Medicine of Tunis, University of Tunis El Manar, Tunis, Tunisia,; 3 Precision Medicine, Personalized Medicine and Oncology Investigation laboratory, LR21SP01, Tunis, Tunisia

**Keywords:** Mucinous tumor, borderline ovarian tumor, primary tumor

## Abstract

**Introduction:**

ovarian Mucinous Borderline Tumors (MBT) are characterized by an epithelial proliferation similar to those of well differentiated adenocarcinomas but are distinguished by the absence of stromal invasion. They are often difficult to diagnose histologically. The aim of the work was to specify the pathological and clinical features and to highlight the prognostic of these tumors.

**Methods:**

study was retrospective including 49 cases of primary ovarian MBT, diagnosed at the Patholgy Department of Salah Azaiez Institute from 1992 to 2019.

**Results:**

median age was 48 years old. Histologically, the cases were divided into 34 cases of pure MBT, 13 cases with intraepithelial carcinoma and 2 cases associating an intraepithelial carcinoma with microinvasion. The majority of our cases were classified FIGO I and only one case FIGO III. Sixteen patients received conservative treatment and 30 received radical treatment. The treatment wasn’t specified in three patients. The prognosis was good in the majority of cases. Only one patient had a contralateral recurrence after a follow-up period of three years. There were no significant differences regarding the risk of recurrence and risk factors such as age, gestation, hormonal status, FIGO stage and conservative treatment. We raised this part.

**Conclusion:**

the prognosis of the ovarian MBT is good. However, it is necessary to multiply the samples to avoid missing a carcinomatous focus with an anarchic invasion of the stroma which constitutes a poor prognosis factor. It was changed by these sentences below: the diagnosis of MBT is not easy. Indeed, the distinction of MBT from carcinomas remains the greatest challenge for pathologists. Once this diagnosis is made with certainty, the tumor can be considered to have a good prognosis, especially stage I tumors which are the most common.

## Introduction

Mucinous Borderline Tumors (MBT) or atypical proliferative mucinous tumors of the ovary represent 15% of all mucinous epithelial tumors and 30 to 50% of borderline ovarian tumors [1].

Ovarian MBT was redefined in the edition of the World Health Organization (WHO) 2020 [1]. They currently include tumors exhibiting exclusively gastrointestinal differentiation. Tumors with an endocervical phenotype are now excluded. They are distinguished from mucinous adenocarcinoma by the absence of stromal invasion. They are often difficult to diagnose histologically since, on the one hand, stromal invasion is not always easy to demonstrate and, on the other hand, the indirect criteria for invasion are not unanimously accepted [1]. MBT with intraepithelial carcinoma and with micro-invasion have been described. Mural nodules which are identified macroscopically may be associated with these tumors [1,2]. The interest in these tumors is justified by their relative frequency, their age of onset which is generally ten years earlier than invasive ovarian tumors and their prognosis which is better than that of cancers of the ovary with a survival of 95% at five years, all stages combined [2]. Therefore, a conservative treatment preserving the fertility of patients who are often young and potentially desirous of subsequent pregnancies is authorized.

Our work focuses on MBT of the ovary, histologically documented, collected in the Department of Pathology of the Salah Azaïez Institute over a period of 27 years (1992-2019). We propose in this work to specify the anatomopathological peculiarities of MBT of the ovary allowing them to be distinguished from mucinous adenocarcinoma according to the new WHO 2020 classification and to identify the prognostic factors.

## Methods

**Study design:** we conducted a retrospective descriptive study on a series of patients with MBT of the ovary whose diagnosis was confirmed on the operative specimen.

**Study location and setting:** all cases were collected in the pathology department of a cancer center “Salah Azaiez Institute” (ISA) in Tunis. Tunis is the capital of Tunisia located in the north of the country.

**Study participants:** all cases were collected over a period of 27 years, from January 1992 to February 2019. We included in our study all patients who presented with a primary MBT on a surgical specimen. Their diagnosis was made in the pathology department. Their evolution was determined from the date of the operation until the date of the last consultation and the latest news for patients lost to follow-up. The collection of clinical data was made from the medical records. Patients whose file was not found, empty or incomplete and patients who were treated for Borderline tumors other than mucinous (serous) were not included in this study. Seromucinous tumors (endocervical type) that were considered before 2014 as MBT of ovary were excluded from our study.

**Data sources and measurement:** electronic medical records and pathology reports were reviewed to analyze clinical parameters (age, parity, hormonal status, clinical presentation, abdominal imaging, tumor markers), pathologic variables (gross appearance, tumour size and histological features). The International Federation of Gynecology and Obstetrics (FIGO) Stage was based on clinical and pathological data.

**Other clinical and follow-up data were also noted:** treatment, recurrence, sites of recurrence and survival.

**Review of the slides was performed to confirm the diagnosis and reassess the following histological parameters:** the architecture of tumor proliferation, the degree of epithelial stratification, cytonuclear grade (grade 1: mild atypia, grade 2: moderate atypia and grade 3: marked atypia), mitotic index (low: < 5mitoses / 10 at high power field (HPF), moderate: between 5 to 9 mitoses / 10 HPF and high: > 10 mitoses / 10 HPF), micro-invasion and intraepithelial carcinoma. The micro-invasion is defined as a stromal invasion of less than 5 mm in the largest linear dimension and regardless of the number of focal points. The invasive cells are either single isolated cells or arranged in glands or in clusters. The atypia is mild to moderate. Intraepithelial carcinoma is characterized by foci of marked nuclear atypia, without a sign of invasion. The presence of a stratification greater than three layers or of a cribriform architecture is insufficient to qualify intraepithelial carcinoma if the atypia is not marked. Atypia usually show a sharp demarcation from adjacent cells.

**Bias:** the retrospective nature of the study did not allow optimal control of the data collected.

**Study size:** a total of 49 cases with confirmed diagnoses of MBT were included in our study.

**Variables:** for the qualitative variables, we calculated the simple frequencies and the relative frequencies (percentages). For the quantitative variables, we calculated the means, the medians and determined the range (extreme value = minimum and maximum). We removed this part!

**The quantitative variables were:** age, parity, CA125 and tumor size. The qualitative variables were macroscopic study, histological type, FIGO stage and treatment.

**Statistical analysis:** the data were entered on an Excel file. For the qualitative variables, we calculated the simple frequencies and the relative frequencies (percentages). For the quantitative variables, we calculated the means, and the medians and determined the range (extreme value = minimum and maximum). The follow-up times of patients were calculated from the date of diagnosis until the date of last follow-up. Statistical analysis was performed using SPSS version 22 software. The chi-square test was used for the comparison of qualitative variables. The links between two quantitative variables were studied by the Pearson correlation coefficient. The value of P <0.05 was considered statistically significant. We removed this part !

**Ethical considerations:** we declare the absence of conflicts of interest and the respect of ethical considerations in our work. We received ethical approval for this study from the ethics committee of Salah Azaiez Institute.

**Consent:** we obtained written informed consent from the patients to publish the details of this article.

## Results

**Participants:** a total of 49 cases with a confirmed diagnosis of ovarian MBT were included in our study. These tumours were present in 25% of borderline ovarian tumors in the pathology department of the Salah Azaiez Institute.

**Descriptive data:** in our series, the patients were between 16 and 87 years old with an average of 48 years old. The median age was 46 years old. The average parity of our patients was around 3.57. Parity was not specified in seven of our patients. Nine patients were nulliparous (21% of cases). Twenty patients were postmenopausal at the time of diagnosis. The circumstances of discovery of the tumours were more often an increase in abdominal volume associated with pelvic pain. Forty patients had an abdominopelvic ultrasound in which the average tumor size ranged from 5 to 31 cm. Computed tomography was performed on 18 patients to determine the origin and extent of the abdominopelvic tumor. The CA125 assay was performed only in 27 patients; the level was high (> 35 U/ml) in 11 cases. The macroscopic study revealed that the median tumour size at the time of surgical resection was 22 cm (range, 6-40 cm). The outer surface was smooth in all cases. A multilocular aspect was predominant, found in 40 cases (82%). Intracystic adenoids were noted in nine cases (18%) and a mixed appearance with cystic and solid areas was observed in 12 cases (24%). The microscopic study revealed 34 cases of pure MBT, 13 cases of MBT with intraepithelial carcinoma and two cases of MBT associating an intraepithelial carcinoma with micro-invasion. The pure MBT were essentially made up of large cystic cavities of variable size, within which were projected filiform and/or pseudo-villous structures provided with a conjunctive vascular axis. They were associated with small to dilated glandular structures, sometimes packed against each other. The prominent papillary architecture was found in 15 cases. The epithelium was mucinous containing goblet cells, gastric cells and endocervical cells in varying proportions. Paneth cells were present in three cases. The stroma showed a non-specific inflammatory infiltrate in six cases, extravasation of mucin with histiocytic reaction from the ruptured cysts in 10 cases, small calcifications in eight cases and foci of lutheinization in two cases.

**Figure 1:**
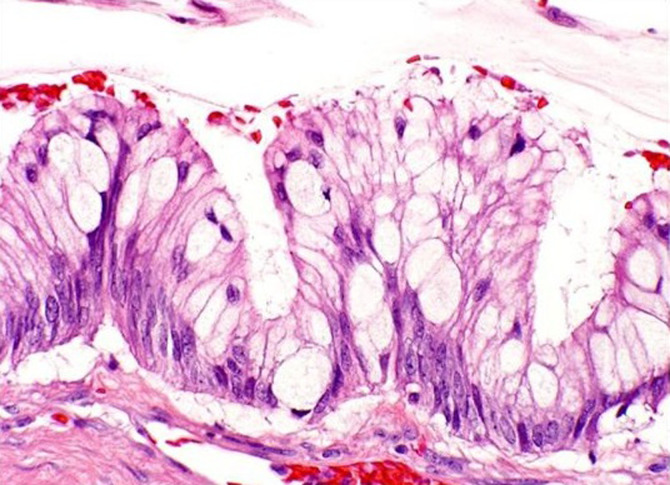
microscopic appearance (X400): epithelial coating with tendency to stratification, the nuclei are moderately irregular in appearance, tumor cells exhibit a mucosecretion of the goblet cell type

**Figure 2:**
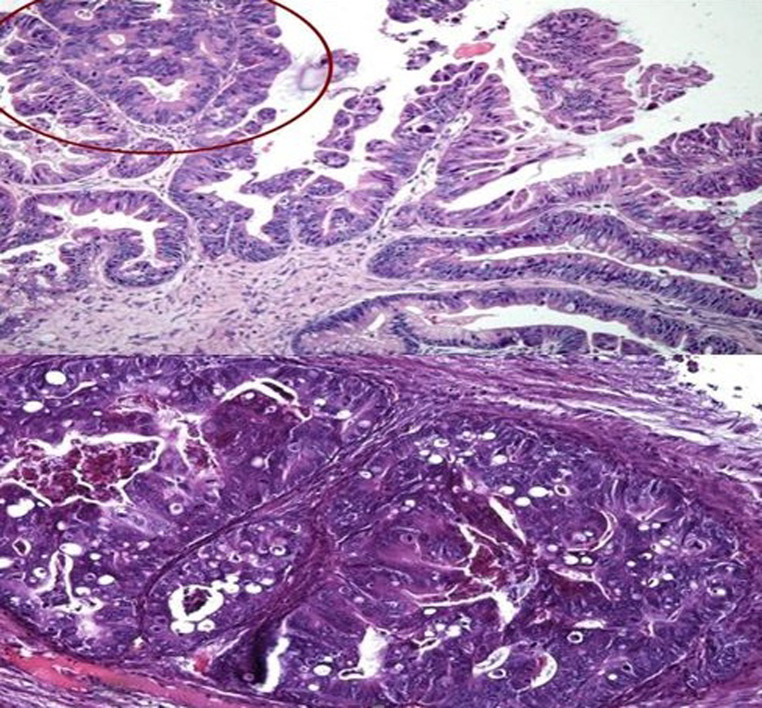
microscopic appearance (X200): sector of carcinoma in situ

An extensive smooth muscle metaplasia was found in two cases. Large intraglandular necrosis with acute inflammatory reaction was present in five cases. Residual ovarian tissue showed mucin extravasation leading to ovarian pseudomyxoma in seven cases. The nuclear atypias were mild to moderate. The mitotic index was low. Figure 1 shows the microscopic appearance of a pure MBT. Figure 2 shows a microscopic appearance of a sector of carcinoma in situ.

**Figure 3:**
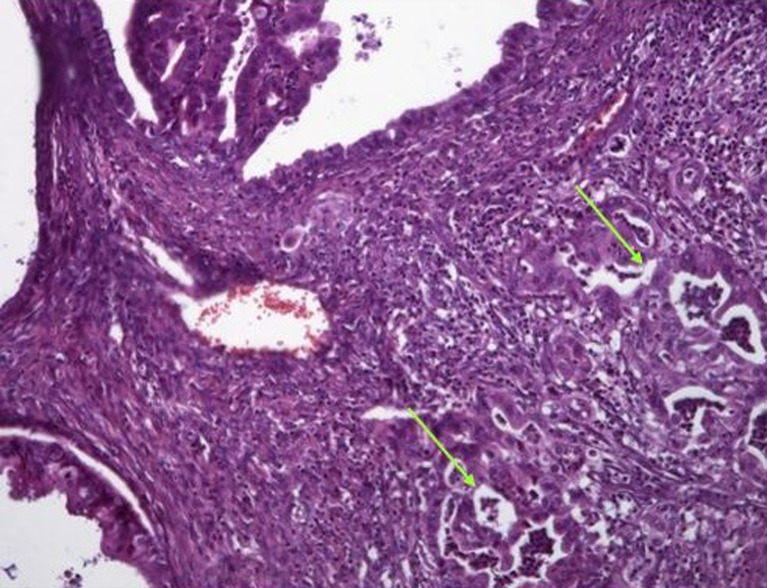
microscopic aspect (X400): Micro-invasion aspect (according to the arrows)

Two cases of MBT with micro-invasion were identified: - in one case, the outbreaks were six in number, each measuring 1 to 2 mm in long axis, made up of isolated cells, small nests and more rarely small irregular glands surrounded by desmoplastic stroma. Nuclear atypias were moderate (G2) without associated mitosis (Figure 3). - The other case has one focus on micro-invasion of a 1 mm long axis made of tubes and isolated cells. The nuclei were inconsistently atypical and focally mitotic. For associated lesions: One case of endometriosis within an ipsilateral ovarian cyst was noted. Endometriosis in the contralateral ovary and on the hysterectomy patch (adenomyosis) was also noted in one case and two cases, respectively. A serious cystadenoma of the contralateral ovary was noted in one case. A mucinous cystadenoma was noted in the contralateral ovary in one case. In two cases, we noted the presence of a Brenner tumor in the ipsilateral ovary. Two cases of MBT were associated with peritoneal pseudomyxoma. One of the two cases developed in a mature teratoma with a normal appendix.

**Treatment:** forty-five tumors were diagnosed and operated on stage I of FIGO: forty-three patients with stage IA and two patients with stage IB. One patient was in stage III. The surgical procedure, in our study, consisted, in 16 patients under 40 years old, of unilateral adnexectomy (+/- appendectomy +/- omentectomy) or cystectomy. Thirty patients underwent radical treatment: total hysterectomy with unilateral or bilateral adnexectomy (+/- appendicectomy +/- omentectomy). Lymph node dissection was performed in three cases. The treatment and FIGO stage weren´t specified in the three patients. Accidental tumor rupture intraoperatively occurred in one case. Chemotherapy was indicated in three patients and it was given (9 cycles over one year) in only one patient who presented with peritoneal pseudomyxoma. The two other patients were lost to follow-up.

### Outcome data

**Clinical outcomes and survival analysis:** we only had postoperative follow-up for 29 patients. The survival rate was over 95%. The follow-up period ranged from three months to 72 months (6 years) after surgery with a mean follow-up of 32 months. Among the 16 patients who had conservative treatment, only one patient had a contralateral recurrence after a follow-up period of three years. She had a left adnexectomy as a second step. She was referred to gastrology on suspicion of peritoneal carcinoma, then lost to follow-up. For the two cases with peritoneal pseudomyxoma, one patient had a favorable outcome after a six-year follow-up, and for the other patient, we have no data on its outcome. We did not have sufficient data to estimate five-year survival.

**Main results:** in our study, we looked for risk factors for recurrence, but we did not find any significant difference: age (P = 0.68), pregnancy (P = 0.83), hormonal status (P = 0.91), and FIGO stage (P = 0.93). Also, there was no correlation between conservative treatment and tumor recurrence (P = 0.69). We removed this part! Demographics and clinicopathologic characteristics of patients with MBT are summarized in Table 1. Median age was 48 years old. Histologically, the cases were divided into 34 cases of pure MBT, 13 cases with intraepithelial carcinoma and two cases associating an intraepithelial carcinoma with microinvasion. The majority of our cases were classified FIGO I and only one case FIGO III. Sixteen patients received conservative treatment and 30 received radical treatment. The treatment wasn´t specified in three patients. The prognosis was good in the majority of cases. Only one patient had a contralateral recurrence after a follow-up period of three years.

**Table 1:** demographics and clinicopathologic characteristics of patients with mucinous borderline tumors

Parameters	N (%)
**Age at diagnosis (years)**	
[15-20]	2 (5%)
[20-30]	4 (8%)
[30-40]	7 (14%)
[40-50]	14 (29%)
[50-60]	9 (18%)
[60-70]	7 (14%)
[70-80]	5 (10%)
> 80	1 (2%)
**Histological type**	
Pure MBT	34 (69%)
MBT with intraepithelial carcinoma	13 (27%)
MBT with intraepithelial carcinoma and micro-invasion	2 (4%)
**FIGO stage**	
IA	43 (88%)
IB	2 (4%)
III	1 (2%)
Not mentioned	3 (6%)
**Treatment**	
Conservative	16 (33%)
Radical	30 (61%)
Not mentioned	3 (6%)
**Recurrence**	
Yes	1 (2%)
No	28 (57%)
Not mentioned	20 (41%)
**Follow up time (years)**	
[0.3-1]	7 (14%)
[1-2]	2 (4%)
[2-3]	8 (16%)
[3-4]	2 (4%)
[4-5]	2 (4%)
[5-6]	6 (12%)
6	2 (4%)
Not mentioned	20 (41%)

ABREVIATION: MBT indicates Mucinous Borderline Tumors; FIGO indicates International Federation of Gynecology and Obstetric

## Discussion

### Summary of main findings

In our series, the average age of the patients was 48 years. MBT were, in more than 90% of cases, unilateral, often voluminous with an average size of 22 cm. They were multilocular cystic surface external smooth generally without vegetations, macroscopically simulating a benign cystadenoma. MBT were typically composed of cysts and multiple structures of glandular cells lined by an intestinal-like epithelium containing cells goblet. Nuclear atypia was mild to moderate. The stroma was poorly developed, often reduced to thin fibrous septa. Thirteen cases had sectors of intraepithelial carcinoma (26% of all the tumors in our series). Intraepithelial carcinoma was characterized by areas of marked nuclear atypia which differed cytologically from background epithelium, usually with a sharp demarcation. Indeed, the diagnosis of intraepithelial carcinoma should be based solely on the nuclear cytomorphology although intraepithelial carcinoma is often associated with architectural complexity with epithelial stratification or growth cribriform; these architectural criteria are neither necessary nor sufficient. Micro-invasion, defined by a stromal invasion of less than 5 mm in the largest linear dimension, was observed in two cases in our series. The treatment of MBT is surgical. It is discussed according to the intraoperative staging of the FIGO classification with systematic verification of the appendix.

**Two options are possible:** radical treatment and conservative treatment, in order to preserve the fertility of patients who are generally young and wishing for pregnancy. 16 patients had conservative treatment and 30 patients received radical treatment. The postoperative follow-up period varied between 3 and 72 months (6 years) with average follow-up of 32 months. The overall survival rate was greater than 95%. The prognosis of stage I MBT with intraepithelial carcinoma and/or micro-invasion was excellent with a 5-year survival greater than 95%. Among the patients who had conservative treatment, only one patient had a contralateral recurrence after a follow-up period of 3 years.

### How the findings relate to previously know the literature

The MBT are the second most common type found and account for approximately 10-15% of ovarian tumors in general and 35-45% of borderline ovarian tumors [1,3]. In our department, these tumours were present in 25% of borderline ovarian tumours. There is great variability in the age of onset of MBT with an interval ranging from 14 to 89 years [4,5]. The average age is 49 years old [4]. The mean age in our series, 48 years, was comparable to that reported in the literature [4-6]. More than half of patients with MBT are nulliparous [7]. In our study, 21% of patients were nulliparous. The clinical presentation of these tumors is nonspecific, dominated by an increase in abdominal volume observed in 29% of cases and pelvic pain in 27.5% of cases [8]. The dosage of CA 19-9 has been proposed in the management of ovarian MBT by analogy with digestive mucinous tumors. CA125 was higher in serous tumors than in mucinous tumors (50.6% vs. 35.5%), while CA19-9 was higher in mucinous tumors than serous tumors (33.6 vs. 15, 3%) and it is currently the most precise tumor marker for the early detection of postoperative recurrence of ovarian MBT [9]. In our series, the level of CA125, evaluated in 27 cases, was high in 41% of cases.

The macroscopic study reveals that ovarian MBT are, in more than 90% of cases, unilateral [2,3,6,10]. They are distributed at equal frequency on the right and on the left. These tumors are often large with an average size of 16.6 cm and extremes ranging from 4 to 32 cm [5,6,11]. Typically, these tumors are multilocular in 72% of cases [4,12,13] producing large cystic masses with a smooth external surface simulating a benign cystadenoma [14,15]. These cystic cavities vary in size, filled with thick, greenish mucoid material. They are often deformed by reciprocal pressure and separated by fine fibrous septa [14,16]. However, the wall of these cavities is sometimes thickened and velvety [10,17,18]. The papillary structures or intracystic adenoids, commonly observed in endocervical-type tumors and in serous tumors, are rarely observed, not exceeding 10% in the series of Lee and Scully [5] and 19% in the series of Rodriguez and Prat [6]. In our series, the macroscopic appearances were similar to those described in the literature with an average tumor size of 21.85 cm and a multilocular appearance observed in 82% of cases. A thorough examination and careful sampling are mandatory and at least one section per centimeter of the largest tumor diameter should be performed, increasing to two blocks per centimetre of diameter in mucinous tumors larger than 10 centimetres [1,2]. We removed this part The microscopic appearances in our study were similar to those in the literature.

The ovarian MBT are typically composed of cysts and multiple glandular structures of unequal size, separated by an ovarian stroma which is usually poorly developed. The epithelium exhibits varying degrees of stratification and filiform papillae [5,6]. Cribriform architecture does not exist in pure MBT [5,6,19]. The areas of proliferation must comprise more than 10% of the epithelial volume of the tumor to be classified as MBT [5,6]. The cysts are lined with a mucinous epithelium of gastric or intestinal differentiation, with papillary or pseudo-papillary folds, and goblet and neuroendocrine cells [20]. The nuclei are basal, isomorphic and with uniformly distributed chromatin [2,13,21]. Nuclear atypias are mild to moderate. The stroma has an often inconspicuous inflammatory infiltrate. It is mainly accentuated in large tumors [10]. Necrosis is not synonymous with malignancy; it is present focally, in almost half of cases [6], or even extended in large tumors [5,10]. The appearance of pseudomyxoma ovarii (cell-free pools of mucin in the stroma) is present in approximately 20% of tumors [1,5] and a granulomatous stromal response to mucin caused by rupture of the gland is common [1,5,6,16]. Histogenetically, the development of MBT from a mature teratoma or via metaplastic Brenner tumors has been discussed and ovarian and Brenner mucinous tumors may share common stem cell differentiation [2, 22-25]. In our work, we found two cases of Brenner tumor associated with a mature teratoma.

Borderline mucinous tumors are characterized by their non-Mullerian differentiation with the absence of WT1, estrogen and expression of progesterone receptors [26,27]. Most tumors demonstrate diffuse expression of cytokeratin 7 with uneven coexpression of cytokeratin 20 and variable, generally low expression, CDX2 in approximately 40% of cases [20,28,29]. Despite limited previous studies that only assessed PAX8 expression in mucinous carcinomas [30], a recent study confirmed PAX8 expression in MBT as well as ovarian mucinous carcinoma [2].

Overexpression of HER2 has been reported in up to 20% of MBT [31] and may sometimes be useful in distinguishing primary ovarian damage from secondary ones. The rate of proliferation is generally low, and Ki67 shows predominant expression in cells at the base of papillary structures [2]. Borderline mucinous tumors with intraepithelial carcinoma have been described in 40 to 55% of MBT and are characterized by areas of marked nuclear atypia that differ cytologically from the background epithelium, usually with a clear demarcation [2,5,19,32]. Although intraepithelial carcinoma is often associated with increased architectural complexity of epithelial stratification or cribriform growth, this criterion is neither necessary nor sufficient, and the diagnosis of intraepithelial carcinoma should be based solely on nuclear cytomorphology. We removed this part! Although some studies have reported a higher risk of recurrence, most studies have found no difference in overall survival for MBT with or without intraepithelial carcinoma [2,5,6,33]. MBT with micro-invasion are defined by stromal invasion less than 5 mm in the longest linear dimension and consisting of single cells, clusters or small foci of glandular or cribriform growth, regardless of the number of micro-invasive foci. Cases with micro-invasive foci presenting high-quality nuclear atypia are classified as micro-invasive carcinoma according to the WHO classification, although the prognostic value of this category remains to be defined [1,15]. We removed this part!

Microinvasion has been reported in 4-18% of MBT and has no negative effect on the prognosis [5,19,32]. Mural nodules which are identified macroscopically may be associated with MBT or carcinomas. Three varieties of nodules have been described, including pseudosarcomatous reaction wall nodules, carcinomatous nodules, and sarcomatous nodules [34-37]. The nodules can be up to about 10 cm in size and they can be single or multiple and sharply demarcated from the adjacent epithelial lining. The reactive nodules are typically hemorrhagic nodules and the neoplastic nodules are solid and white. Pseudosarcomatous nodules show a heterogeneous cell population with numerous multinucleated cells, atypical spindle cells and inflammatory cells. In some nodules, the predominant elements are spindle-shaped cells of moderate size with hyperchromatic nuclei, pleomorphic mononuclear cells, and binucleate giant cells. The mitotic index in most cellular areas is often high [1].

Sarcoma-like wall nodules generally show weak or focal cytokeratin labeling [36]. Their circumscription and large cellular inflammatory component suggest that they represent a reaction to hemorrhage or mucinous contents of cysts [36]. We removed this part! In our study, we did not find any wall nodules. Strengths and weakness During this work, we encountered some methodological difficulties, mainly due to the retrospective nature of our study such as the lack of precision of some information at the file level medical and operative reports. Microscopic examination was based on morphology and immunohistochemistry was not used because of its high cost. We did not have sufficient data to estimate five-year survival. We studied the general characteristics of the population, the aspect of macroscopic and microscopic tumors, the surgical treatment and patient follow-up. So, the strengths of this work are that this data adds to the body of knowledge of a rare tumor in a cancer reference center in tunisia, a country in northern Africa. This work would be interesting to physicians who may be the first to see the patients all the way to general surgeons, gynaecologists and gynaecology oncologists.

### Recommandations based on findings, future work to be explored

The MBT diagnosis is a challenging diagnosis that must exclude first a benign mucinous cystadenoma and second a primary or secondary mucinous carcinomatous tumor of the ovary. The association of a benign component within MBT has been frequently reported [15,38]. The presence of a small borderline component in a cystadenoma not exceeding 1 to 2% of the tumor surface has no prognostic impact and does not classify this tumor in the category of borderline tumors. The borderline component must occupy, according to WHO recommendations, more than 10% of the tumor surface to classify the tumor as borderline [1]. The extra-ovarian locations of MBT, reported in 12% of cases [5], are exclusively represented by lesions of peritoneal pseudomyxoma or gelatinous disease of the peritoneum [39-41].

Tumors associated with peritoneal pseudomyxoma lesions are more frequently bilateral, almost always including areas of ovarian pseudomyxoma and often associated with appendicular lesions [39,42,43]. According to the data in the literature, in the event of such associations, the appendicular location is most often the primary lesion; ovarian and peritoneal locations are secondary [15,43]. In our work, two patients presented with peritoneal pseudomyxoma with a healthy appendix. The prognosis for these tumors is excellent; only few cases of progression to carcinoma have been reported and these tumors have not been sufficiently sampled. Therefore, the possibility that occult areas of carcinoma were missed cannot be excluded [1]. The survival rate for these stages I tumors, without intraepithelial carcinoma and without micro-invasion, is around 95%. The five-year mortality rate is 1% and the recurrence rate is 1.8% [1,3,8,44]. The overall survival of women with MBT with intraepithelial carcinoma is 95% to 100%. Rare cases of death have been reported from late stage tumors [5,44]. The recurrence rate observed in these tumors is slightly higher than that in low tumor mutational burden (TMBL) without intraepithelial carcinoma. Recurrence has been reported in almost 6% of cases of a total of 208 MBT with intraepithelial carcinoma stage I [5,10]. The recurrence rate of MBT with micro-invasion is 5% and the tumor-related mortality rate is less than 5% [1,33]. The association with peritoneal pseudomyxoma almost always leads to recurrence. The five-year death rate is approximately 50% [3,45]. Several prognostic factors for relapse have been identified. Younger age, advanced FIGO stage (FIGO stages II - IV) and invasive implants appear to be the most significant and consistent prognostic factors for relapse proven to date [46,47]. In a Danish series, other prognostic factors were identified such as laparoscopy, fertility-preserving surgery, and microinvasion [44]. In our study, we had only one case of recurrence for 16 patients who had conservative surgery. We did not have sufficient data to estimate five-year survival. Moved above! Surgery is the main method of treating these tumours. We removed this sentence! Finally, as described in the previous section, emerging knowledge supports the notion that subtypes of borderline ovarian tumors comprise distinct biologic, pathogenetic, and molecular entities which are being discovered [2].

## Conclusion

At the end of this work, we conclude that the distinction of TMBL from carcinomas, in particular metastatic, remains the greatest challenge for pathologists. Once this diagnosis is made with certainty, the tumor can be considered to have a good prognosis, especially stage I tumors which are the most common. The presence of intraepithelial carcinoma does not appear to influence the prognosis. However, more samples must be taken so as not to miss a carcinoma outbreak with anarchic invasion of the stroma, which is a factor of poor prognosis. Changed! We conclude that the diagnosis of these rare TMB tumors is not easy. Indeed, the distinction of TMBL from carcinomas remains the greatest challenge for pathologists. Once this diagnosis is made with certainty, the tumor can be considered to have a good prognosis, especially stage I tumors which are the most common. So, it is necessary to recognize the different histological particularities. Prospective and multicenter studies (to have a larger number of patients) would be necessary for a better understanding of these tumors and their evolution.

### What is known about this topic

- The ovarian MBT are often difficult to diagnose histologically;
- The prognosis of ovarian MBT is better than that of cancers of the ovary with a survival of 95% at five years, all stages combined.

### What this study adds

- The pathological and progressive characteristics of ovarian MBT according to the definition of the edition of the World Health Organization (WHO) 2020;
- Highlights on micro-invasion, carcinoma in situ...in MBT;
- There is no correlation between conservative treatment and tumor recurrence.
